# Ultra-Low-Voltage Capacitive Micromachined Ultrasonic Transducers with Increased Output Pressure Due to Piston-Structured Plates

**DOI:** 10.3390/mi13050676

**Published:** 2022-04-26

**Authors:** Fabian Merbeler, Sonja Wismath, Marco Haubold, Christian Bretthauer, Mario Kupnik

**Affiliations:** 1 Measurement and Sensor Technology Group, Technische Universität Darmstadt, Merckstr. 25, 64283 Darmstadt, Germany; sonja.wismath@tu-darmstadt.de (S.W.); mario.kupnik@tu-darmstadt.de (M.K.); 2 Infineon Technologies AG, Am Campeon 1-15, 85579 Neubiberg, Germany; christian.bretthauer@infineon.com; 3 Infineon Technologies Dresden, Königsbrücker Str. 180, 01099 Dresden, Germany; marco.haubold@infineon.com

**Keywords:** capacitive micromachined ultrasonic transducer (CMUT), surface micromachining, ultra-low voltage, piston-like movement, high-efficiency output pressure

## Abstract

Capacitive micromachined ultrasonic transducers (CMUTs) represent an accepted technology for ultrasonic transducers, while high bias voltage requirements and limited output pressure still need to be addressed. In this paper, we present a design for ultra-low-voltage operation with enhanced output pressure. Low voltages allow for good integrability and mobile applications, whereas higher output pressures improve the penetration depth and signal-to-noise ratio. The CMUT introduced has an ultra-thin gap (120 nm), small plate thickness (800 nm), and is supported by a non-flexural piston, stiffening the topside for improved average displacement, and thus higher output pressure. Three designs for low MHz operation are simulated and fabricated for comparison: bare plate, plate with small piston (34% plate coverage), and big piston (57%). The impact of the piston on the plate mechanics in terms of resonance and pull-in voltage are simulated with finite element method (FEM). Simulations are in good agreement with laser Doppler vibrometer and LCR-meter measurements. Further, the sound pressure output is characterized in immersion with a hydrophone. Pull-in voltages range from only 7.4 V to 25.0 V. Measurements in immersion with a pulse at 80% of the pull-in voltage present surface output pressures from 44.7 kPa to 502.1 kPa at 3.3 MHz to 4.2 MHz with a fractional bandwidth of up to 135%. This leads to an improvement in transmit sensitivity in pulsed (non-harmonic) driving from 7.8 kPa/V up to 24.8 kPa/V.

## 1. Introduction

After their invention in 1994, capacitive micromachined ultrasonic transducers (CMUTs) arose as a new and promising type of ultrasonic transducer for various applications [1]. Generally, CMUTs consist of a fixed backplate, a movable plate electrode and a vacuum gap in between. Such transducers provide high operating frequency range as well as ease of fabrication and integrability with established CMOS processes and technologies, allowing for both small and large devices due to excellent scalability. This makes this type of microelectromechanical systems (MEMS) one of the most promising candidates for advancing the state of the art in many fields, such as high-intensity focused ultrasound (HIFU) therapy [2], medical imaging (e.g., 3D imaging [3], intravascular ultrasound (IVUS) imaging [4,5,6], intracardiac echocardiography (ICE) imaging [7], and strongly emerging photoacoustic imaging [8,9]), and ultrasound computer tomography [10]. Furthermore, diverse industrial applications, such fill-level sensing [11] and flow-metering [12], structural health monitoring [13,14], resonant gas sensing [15,16,17], and consumer applications (e.g., fingerprint sensors) [18], are advanced due to CMUTs. A comprehensive review on CMUTs is given by Brenner et al. [19].

Compared to common ‘bulk piezo’ transducers and piezoelectric micromachined ultrasonic transducers (PMUTs), CMUTs have better electro-mechanical coupling, improved fractional bandwidth (fBW), and avoid piezoelectric materials, which are expensive, have higher thermal noise, and are difficult to control in fabrication.

Two major drawbacks of established CMUT technology are the need for high (bias) voltages and the limited output pressure compared to, for example, single-crystal piezoelectric bulk transducers, as CMUTs rely on a fixed gap between the electrodes limiting the displacement possible. The CMUT design presented in this paper directly addresses these two challenges. Low-voltage operation is enabled via both thin plate height and thin gap height. This implies decreased mechanical resistance and increased electromechanical coupling, respectively. Thereby, our design fulfills the low voltage requirements for integration in established technologies, with charge pumps providing up to 24 V. Additionally, the circular movable plate features a circular piston area for stiffening the upper side. This geometry significantly improves the average displacement due to a more piston-like movement, and thus the sound pressure emitted is strongly increased. A sound pressure of 500 kPa is a requirement for decent signal, especially when targeting sound emission and propagation in materials of high acoustic impedance or multi-layer systems. We aim for operation in the low MHz range for less loss in propagation due to attenuation compared to higher frequencies. In this paper, we show finite element method (FEM) simulations accompanied by characterization measurements for mechanical properties, pull-in voltage, and acoustic performance. Three designs are presented for comparison. The operational frequency is in the low MHz range (<4.5 MHz), pull-in voltages are <25 V, and the acoustical performance is increased by a factor of 11 to 500 kPa only via the addition of the piston compared to the bare plate.

### 1.1. State of the Art

In this section, we present a literature review for different approaches of adapted CMUT plate geometries with the goal of enhancing the emitted sound pressure. An overview of key figures is given in Table 1.

In reference [20], the authors designed a piston CMUT with a dual electrode system with surface micromachining technology. The plates and pistons of the cells are rectangular, with the center mass (piston) extending over 50% of the width and almost over the entire length of the plate. In addition, different plate thicknesses and piston thicknesses are compared. The coupling coefficient and transmit bandwidth (BW) show an improvement from uniform (non-piston) to non-uniform (piston) design. Thereby, the coupling coefficient $k^{2}$ increases from 0.28 to 0.40 for the standard electrode design. The increase in fBW is expected to be from 107% to 137%, as confirmed by hydrophone measurements. However, the pull-in voltages in this reference range from 80 V to 160 V, which is relatively high for mobile battery-driven applications. Further, the plate material thicknesses for both electrode designs are varied from 3.5 μm without piston to 2.75 μm with piston, impeding a comparison of the mere effect of the piston.

Moreover, CMUTs with pistons placed on the bottom of the movable plate within the gap are presented by Huang et al. [21]. The squared cells are fabricated with wafer-bonding technology and have pull-in voltages of 47 V without piston and 65 V with piston. The team reports improvements due to the piston in transduction efficiency (transmission and reception) and BW as well. The transmit performance improvement is about 80%, from 3.15 kPa/V_AC_ to 5.57 kPa/V_AC_, while receive sensitivity increases by 95%, from $4.23\;V_{AC}/kPa$ to $8.25\;V_{AC}/kPa$, due to the addition of a piston. The fBW increases from 110% to 150%. However, the compared transducers have different edge lengths of squared cells. The piston transducer cell is 120 × 120 $\mu m^{2}$ with a 70 × 70 $\mu m^{2}$ piston and a gap of 440 nm compared to the cell with no piston of 88 × 88 $\mu m^{2}$ and a gap of 850 nm. This gives an overall advantage to the piston transducer design that relates to the interfering effects of a larger plate, smaller gap, and the presence of a piston.

**Table 1:** Overview of CMUT properties of state-of-the-art designs for enhanced ultrasound output.

	Lateral Geometry (μm)	Gap, Insulator (nm)	Sound Pressure (MPa)	Pull-In Voltage (V)	Technology	Center Frequency (MHz), fBW (%)
Guldiken et al. [20]	Rectangular, a/d = 50% (width)	120, 250	NA	129	Surface micromachining	8, 136 (3 μm parylene coating)
Huang et al. [21]	Squared, a/d = 58%	440, NA	1.28	65	2× wafer bonding	2.5, 150
Wong et al. [22]	Circular, a/d = 80%	400, 600	1	140	Wafer bonding	3, NA
Jeong et al. [23]	Non-trivial, ind. clamped	350, NA	NA	95	2× wafer bonding	3.37, NA
Lee et al. [24]	Non-trivial, posts in substrate	150, 200	1.88	78	Wafer bonding	1.8, 81
Yu et al. [25]	Circular, ring stiff. (PI op.)	300, 2 × 150	3.9 kPa	55	Surface micromachining	6.1, 52.5
Park et al. [16]	Circular, low-voltage design	50, 80	NA	10	Wafer-bonding	7.4, NA
This paper	Circular, piston on-top	120, 25	0.04 to 0.5	7.4 to 25	Surface micromachining	3.3 to 4.2, up to 135

Wong et al. present a CMUT design fabricated with wafer-bonding technology comprising a circularly shaped plate adapted with a piston of 80% plate radius on top [22]. The piston is fabricated with a thin oxide layer and bulk silicon on top. The pull-in voltage is 140 V. Comparing transducers of similar center frequency and pull-in voltage, they report that the piston facilitates an improvement of 30% in transmission, with a surface acoustic pressure of 1 MPa at a bias of 80% of the pull-in voltage. With this sound pressure level (SPL), the authors demonstrated the requirements for HIFU applications of CMUTs.

Jeong et al. present an advanced CMUT geometry to realize a more efficient piston transducer [23]. The authors propose a fabrication process including two wafer-bonding steps (silicon direct bonding and eutectic bonding) to realize an indirectly clamped plate structure. This indirect clamping is achieved with a stiff plate inside the gap, which is mounted to a top membrane with a hole at the center. Thereby, the top plate ring is intended to act as spring, providing the ability to move, while the lower plate in the gap presents the main sound emitting surface performing a piston-like displacement. Although the gap is reduced with the addition of the indirectly clamped plate, the pull-in voltage of the CMUT design demonstrated is still 95 V, while the bare plate without an intermediate mounted plate has a pull-in voltage of 115 V.

In the emerging field of piston CMUT transducers, another approach is presented by Lee et al. [24]. Here, a silicon-substrate-embedded spring system is realized via wafer-bonding technology. The 20-μm-thick plate of 100 μm × 100 μm is supported by nine thin and long rods of Ø4 μm × 50 μm reaching through a gap of 150 nm and 200 nm insulation layer into trenches in the substrate. By that, squared pistons for non-flexural movement are realized, enabling enhanced transmission efficiency. The pull-in voltage is 78 V. The authors achieved 1.88 MPa at the sensor surface with a fBW of 81%.

In [25], Yu et al. present a design with an annular embossed pattern on a plate for pull-in operation at 170 V. A surface micromachining process is adapted to provide an additional nickel ring on a silicon nitride plate. This ring is intended to be located on the movable part during pull-in operation. The authors demonstrate improvements in BW and higher transmission efficiency due to the additional mass ring. From bare plate to embossed plate, the fBW is improved from 41.8% to 52.5%, while the output pressure is improved by 10.2% when operating both CMUTs at the ideal point of bias voltage.

This review of the state of the art reveals the capabilities of enhanced output pressure via nonflexural piston-like movement of CMUT plates. The basic concept is realized by adding geometrical stiffening parts to the plate.

## 2. Design and Fabrication

### 2.1. Design

The innovative CMUT design presented in this work has the goal of providing a low-voltage operation for good CMOS integration, and at the same time ensuring an output pressure with an MPa range in the low MHz range. With such sound pressure intensities and frequencies, the design enables coupling into, for example, plastics and metals with decent sound propagation. The design further facilitates an operation voltage below 10 V for the bare plate. The small form factor enhances the capabilities for all kinds of space-critical applications. This goal is achieved with a vacuum gap of only 120 nm between the back electrode and the movable plate electrode. Further, a thin, circular, and conductive poly silicon plate acts as sound-emitting surface and top electrode. Due to a small plate thickness of 800 nm, a low spring constant can be achieved, and thus low operational voltages. Furthermore, the addition of a piston geometry improves the average displacement. The stiffening ring is placed on top of the center of the movable plate, providing a piston movement. Thereby, the emitted sound pressure level is significantly enhanced. Additionally, a piston suppresses higher harmonic modes, as reported theoretically by Senlik et al. [26], and shifts the second harmonic resonance frequency to higher values, as simulated by Huang et al. [21]. An improved electro-mechanical coupling coefficient is expected near the pull-in voltage due to the piston geometry as well [20]. Moreover, there is no need for a metal layer on top of the movable plate, as it is conductive itself. This eliminates an additional source of stress due to different coefficients of thermal expansion and generally omits another a material layer.

**Figure 1:**
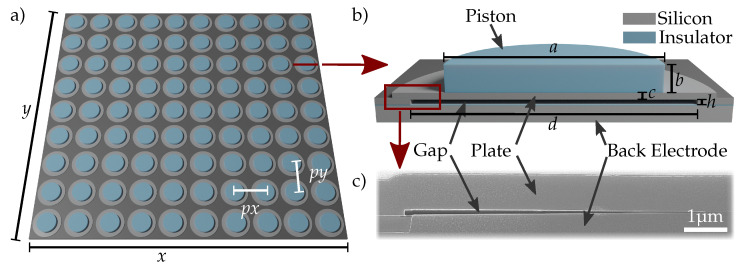
CMUT design with center plate stiffening (piston) on top of a conductive plate. The stiffening is realized with insulating materials (blue), whereas plate and back electrode are made of silicon separated by a thin insulating layer. (**a**) An array of 10 × 9 cells on a roughly squared grid with pitches p${}_{x}$ and p${}_{y}$ forms the active area x-by-y of a single element. (**b**) Schematic representation of the cell cross section (not to scale). The denoted design parameters are given in Table 2. (**c**) SEM image of plate edge with anchor and gap. Left lower corner shows anchor isolation not depicted in (**b**). The plate is in contact with back electrode due to sample preparations for SEM imaging.

As visualized in Figure 1a and listed in Table 2, the CMUT chip comprises 90 plates in a matrix of 9 × 10 on a roughly squared grid (lateral pitch is 61.5 μm and 56 μm). This design presents an active area of $0.3\;mm^{2}$. The small gap amounts to 120 nm in height *h*, whereas the plate has a thickness *c* of 800 nm. Three different designs are fabricated: the plate without a piston, in addition to a piston with diameter *a* of 15 μm, and a piston of 25 μm, allowing us to compare the influence of the piston width on the mechanics of the plate. The circular plate has a diameter *d* of 44 μm, and thus is covered by a piston of an area of 0%, 34%, and 57%, respectively. The piston height *b* is 2.5 μm. An insulation layer of 25 nm is placed on the back electrode to avoid a shortage in case of pull-in.

Compared to the approaches reported in Section 1.1, the introduced CMUT design has the following advantages: the CMUT is more sensitive (TX and RX) compared to a design with a piston inside the gap [21], as the plate can be further deflected by the bias voltage before contact occurs. Thus, the gap between the electrodes is smaller and the sensitivity increases, as analytically described by Wygant et al., that is,

(1) $$\frac{dC}{d\omega_{av}}=\frac{\epsilon_{0}\pi {\left(d/2\right)}^{2}}{2h_{eff}\omega_{av}-6\omega_{av}^{2}}-\frac{C}{2\omega_{av}}\frac{1}{h_{eff}},$$

with cell capacitance *C*, average plate displacement $\omega_{av}$, dielectric constant $\epsilon_{0}$, plate radius *d*/2, and effective gap height $h_{eff}$ [27]. The change in capacitance relative to displacement corresponds to the CMUT sensitivity. Further, the proposed approach of surface micromachining technology is more efficient to fabricate compared to wafer-bonding technologies. The applied technology generally is more cost effective compared to a spring system fabricated into the back electrode/gap [24] or another intermediate plane for an indirectly clamped moving plate [23]. Our technology approach uses established processes and avoids additional process steps. Thereby, it allows the integration of piston and non-piston plates. This can be used, for example, for improved pulse-echo performance with high emission output with piston and high receive sensitivity without piston. Moreover, the presented design allows low-voltage operation of <25 V, compared to the 80 V to 240 V seen in the literature [20,21,22,23,24,25]. Without piston, the design even achieves <10 V, which is especially attractive for mobile battery-driven consumer applications. Another target is to avoid the pull-in operation, further easing the fabrication process, with no need for anti-sticking measures inside the gap. Additionally, we report a CMUT with movable plates of the same dimensions (lateral size and material thickness) with differently sized pistons or no piston, providing insight into the mere effects of pistons, and thereby provide comparability of same-size devices for the first time to the authors’ knowledge.

### 2.2. Fabrication

**Figure 2:**
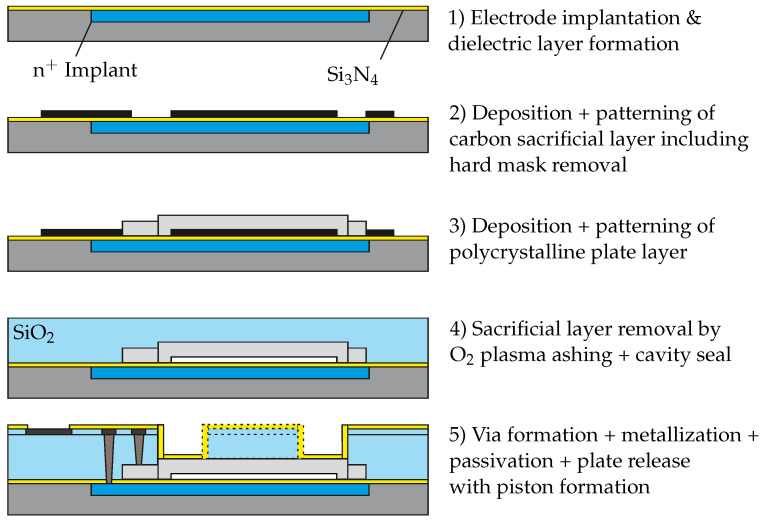
Surface micromachining process flow scheme of CMUT fabrication including a piston structure on top of the movable plate.

The presented CMUT design augmented with a piston is fabricated by surface micromachining, as schematically depicted in Figure 2. The process flow involves phosphorous ion implantation into the silicon substrate to form the back electrode. A subsequently deposited silicon nitride (Si${}_{3}$N${}_{4}$) film assures electrical insulation with respect to the additional transducer components. Thereafter, a hydrogenated amorphous carbon-based sacrificial layer of 120 nm is deposited by plasma-enhanced chemical vapor deposition (PE-CVD). This carbon layer is then patterned using a SiO${}_{2}$ hard mask in combination with photoresist to define the electrode gap. The advantage of carbon material is a dry removal process by means of oxygen plasma ashing, eliminating the risk of sticking by conventional wet release options. Next, a 800 nm amorphous silicon layer is prepared by PE-CVD and plasma etching on top of the carbon film, forming the movable plate of the transducer. A thermal anneal in nitrogen atmosphere at a temperature higher than $600\;{}^{{}^{\circ}}C$ is applied for crystallizing the silicon film and electrical activation of dopants. Furthermore, vacuum sealing by high-density plasma CVD of silicon oxide (SiO${}_{2}$) is utilized for providing a high vacuum level in the cavity and excellent long-term stability. Electrical contacts are formed by vertical vias within a 2.5 μm thick silicon oxide film in combination with a single layer of aluminum to provide signal routing and bonding pads. Following the metallization, the stack is passivated with several 100 nm of silicon nitride. Finally, the dielectrics, such as silicon oxide and passivating silicon nitride, are removed at the active plate area and the electrical contact pads. By modifying the design of the plate release structure, the entire SiO${}_{2}$/Si${}_{3}$N${}_{4}$ volume may be removed to form a large cavity or to fabricate the pistons with specified shape on top of the plate.

## 3. FEM Simulations

The CMUT design was simulated with the help of finite element method (FEM) to gain understanding on the mechanics of a circular plate in combination with pistons. The simulations comprised resonance and pull-in characteristics and allowed for insights into the material properties of the deployed fabrication technology. The analysis was conducted in COMSOL Multiphysics v5.6 (COMSOL AB, Stockholm, Sweden). An overview of simulation results together with the corresponding measurement results is given in Table 3.

**Figure 3:**
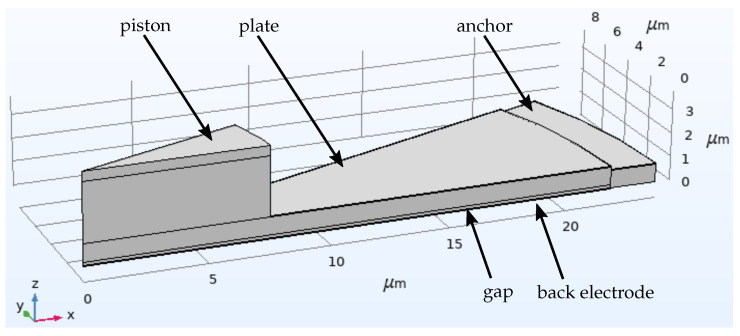
Model geometry in COMSOL. The 3D model uses rotational symmetry on a pie piece of 20 ${}^{\circ }$.

The model geometry is depicted in Figure 3. A 3D model is preferred over a 2D rotationally symmetric one as it is more robust to the non-linearities of the pull-in situation. A pie piece of 20${}^{{}^{\circ}}$ of a single cell was modeled, comprising all material layers. In the physics section, the model was given a symmetry on the lateral surfaces in order to achieve a full revolution of 360°. The atmospheric pressure and initial stress values were accounted here. The mechanical plate resonance was determined with the built-in Eigenfrequency solver, whereas the pull-in voltage simulation took the help of a quasi-static approach. A ‘global equation’ received a static displacement value as input and was solved for bias voltages, leading to this displacement. Thereby, the stable and unstable solutions for the bias voltage $V_{bias}$ below and above the pull-in voltage, respectively, were simulated corresponding to the formula for a parallel plate transducer connected to a spring, that is,

(2) $$V_{bias}\left(z\right)=\sqrt{\frac{2\;\cdot \;k}{\epsilon \;\cdot \;A}}\;\cdot \;\sqrt{\frac{z}{h}}\;\cdot \;\left(1-\frac{z}{h}\right),$$

with displacement *z*, equivalent spring constant *k*, electric permittivity ϵ, area of electrodes *A*, and gap height *h* (compare formula 15.13, p. 227 in [28]). The maximum of the extracted V-z-curve represents the pull-in voltage value of the simulation [28].

### 3.1. Resonance

**Figure 4:**
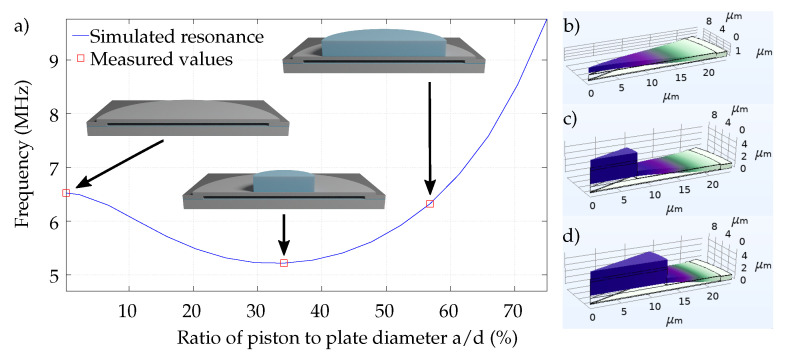
FEM simulation on resonance behavior. (**a**) Resonance frequency in dependence on piston width. The red squares indicate the realized sensors in the presented work. *a* = piston diameter; *d* = plate diameter; (**b**–**d**) Shapes of fundamental mode for plate without and with pistons. The dynamic displacement of the plate is dominated by regions where no piston is on the plate (ring around piston). The piston provides a non-flexural parallel plate movement.

The resonance simulation of the bare plate is depicted in Figure 4a. With rising piston width *a* in relation to the plate width *d*, the resonance frequency decreases from 6.52 MHz down to 5.22 MHz for a/d < 34%. On the one hand, the dominant dynamic mechanical effect in this regime is the addition of mass to the movable plate due to the piston width. On the other hand, the simultaneously expected increase in stiffness is weaker in the dynamic behavior of the plate, and thus the corresponding increase in resonance frequency is not present. However, the effect of an increase of the spring constant dominates at a/d > 34%, and thereby the resonance frequency rises non-linearly. The red markers in Figure 4 indicate the measured values of three fabricated devices and show good agreement in comparison to the FEM simulations, with a deviation of +2.1% and −0.3%. In general, the chosen CMUT design reaches the goal of an operational frequency in the lower MHz range.

### 3.2. Modal Shape of Piston Movement

The key goal of the presented CMUT design is to enable a piston movement. Therefore, the part of the plate covered by the piston is intended to stay flat and is not supposed to bend during movement. The modal shapes of the fundamental mode of the three designs are presented in Figure 4b–d.

### 3.3. Pull-In

**Figure 5:**
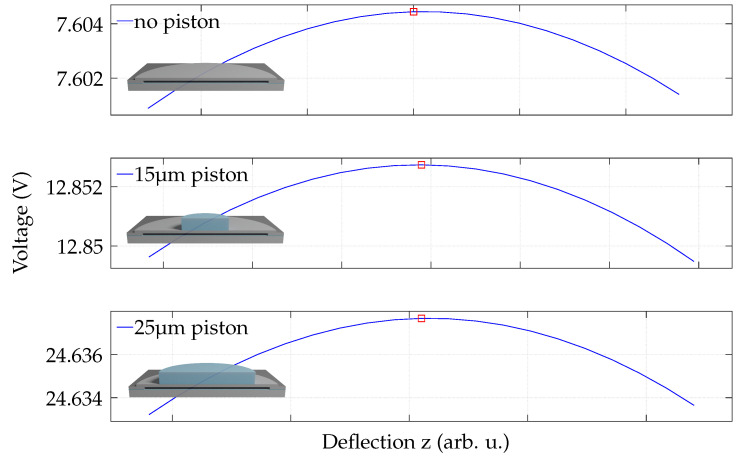
Simulated pull-in voltage of a single cell. The values right to the maximum of each graph are unstable solutions calculated by the quasi-static solver approach. The pull-in voltage is extracted at the maximum of each graph (red markers) for the presented designs.

The pull-in voltage is a crucial property for CMUTs, as it determines the operational point for bias voltage and driving voltage. The simulation results for the pull-in of the three designs are visualized in Figure 5. As described in the methodology section, the maxima (red markers) depict the pull-in voltage. The bare plate has a pull-in of only 7.60 V, whereas a 15 μm piston shows 12.85 V and a 25 μm piston reveals 24.63 V. Moreover, these results indicate that the stiffness of the movable part of the cell increases with piston size, and thus higher voltages are necessary to deflect the plate statically and reach the pull-in state in the presence of pistons. Overall, the simulations are in excellent agreement with the measurements (Table 3). In general, the simulations further show the feasibility of low-voltage operation of this CMUT. When operating below the pull-in voltage, the maximum utilizable voltage is below 25 V. The bare plate is even usable for below 7.6 V, reaching the goal of operation below 10 V as explained in Section 2.1.

## 4. Characterization Measurement Results

### 4.1. Mechanical Characterization

The mechanical properties of the different CMUT designs were evaluated with a laser Doppler vibrometer (LDV)(Polytec MSA-500, Polytec GmbH, Waldbronn, Germany). Thereby, the feasibility and performance of the fabrication process were evaluated to give a proof of concept in addition to the simulations. The laser spot was focused and targeted to the middle of each plate of the 9 × 10 matrix and measures the velocity of the surface via interferometry. The given values represent the average of all 90 cells of a whole CMUT element, respectively. The measurements were performed with a weakly biased CMUT at $2\;V_{DC}$ and white noise of amplitude $1\;V_{AC}$, with an averaging over 10 measurements. The resonance frequency was extracted with a fast Fourier transform (FFT) of the observed plate movement. Furthermore, a single plate was scanned with higher magnification. A grid of measurement points was implemented to extract the modal shape of the plate. The same electric actuation was applied, and measurements were evaluated at the resonance frequency. These measurements for resonance and modal shape were performed under ambient conditions. Moreover, the sensors were characterized in immersion. The actuation was a 100 ns pulse with an amplitude of 80% of the respective pull-in voltage. This was the same actuation as applied in the hydrophone measurements (see Section 4.3). The scan was again set to measure the deflection at the center of each cell for 90 cells. The extracted average center deflection over 90 cells was set against the modal shape to calculate an average deflection of the plates in immersion. Thereby, a peak pressure on the sensor surface could be calculated to be compared against a peak pressure of the hydrophone measurements.

**Figure 6:**
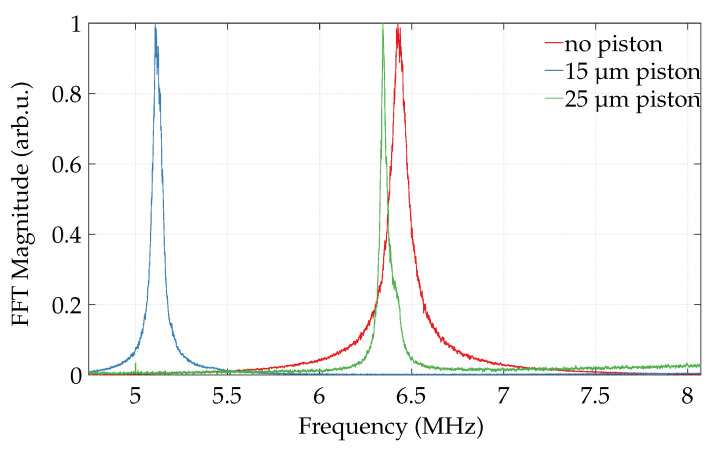
Vibrometer measurements for detecting resonance frequencies of weakly biased ($2\;V_{DC}$) CMUTs with no piston, and with 15 μm and 25 μm wide pistons. Graphs show the normalized average of 90 cells of each design.

The resonance frequency for the plate without piston is 6.43 MHz, as depicted in Figure 6. Further, the plate with smaller piston shows a resonance of 5.11 MHz, while the plate with bigger piston has 6.34 MHz. The quality factors are 81, 106, and 256, respectively. Thus, with the addition of a (bigger) piston, the quality factor rises. Moreover, the standard deviation in frequency across 90 plates of a sensor of each design are 24 kHz, 26 kHz, and 9 kHz, respectively. The piston further improves the fabrication quality and cancels out the sensitivity on process variations. For the biased CMUTs, differently strong spring-softening shifts of the mechanical resonance frequencies are observed.With a voltage step from $2V_{bias}$ to $3V_{bias}$, the shifts amount to 78.5 kHz, 12.1 kHz, and 3.9 kHz, respectively. Thus, the spring softening is negligible in relation to the observed frequency, and the results for each design at the same bias voltage are comparable. These results are in good agreement with simulations in Section 3.1 and support the interpretation given there.

**Figure 7:**
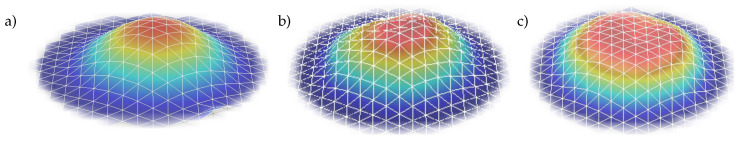
Shape of fundamental mode scanned with a laser Doppler vibrometer. Plates without piston (**a**), with 15 μm piston (**b**), and with 25 μm piston (**c**) are rastered with high magnification.

Moreover, the scans for fundamental mode of the plates are depicted in Figure 7. Here, one can observe an effective stiffening of the plate due to the pistons, as the center part of maximum displacement is extended in diameter by the piston width. The pistons do not show deflection across their diameter. The ratio β of center deflection to average deflection

$$\beta =\frac{\omega_{ave}}{\omega_{center}}$$

is increased substantially with the size of the piston. The bare plate has β=0.30, as expected from theory [27]. The small piston has β=0.40, while the big piston has β=0.49, strongly approaching the pure piston movement with β=1.

The measurements in immersion reveal an average deflection of 2.28 nm, 7.25 nm, and 10.8 nm for the bare plate, and the small and big piston, respectively. This translates into an expected surface pressure p atop a single cell of 3.25 kPa, 13.1 kPa, and 29.8 kPa, respectively, according to

$$p=Re\left(Z_{rad}\right)\;\cdot \;2\pi \;\cdot \;f\;\cdot \;\omega_{average}$$

with radiation impedance $Z_{rad}=\left[0.09,0.11,0.15\right]\;\cdot \;Z_{m}$ at center frequency, respectively, with acoustic (plane wave) impedance of the medium $Z_{m}$ = 0.986 MRayl [19], frequency *f*, and average deflection $\omega_{average}$ (including refractive index correction due to the silicone oil) [24,29]. This is within −6% to +33%, compared to the calculated surface pressure of a single cell in the hydrophone measurements. The deviation stems from several assumptions and neglects, for example, radiation impedance of the specific array, acoustic crosstalk across the surface, piston movement of the plate, and effective radiating aperture of the array.

### 4.2. Electrical Characterization

In this section, the C-V-characteristics of the CMUTs are evaluated. The CMUTs were analyzed with an LCR-meter (Keysight HP4980A, Keysight Technologies, Santa Rosa, CA, USA) on the chip level. The capacitance was determined via applying an AC voltage of 100 mV, with 10 kHz being a far off resonance in air. The DC bias was swept in steps of 100 mV from 0 V to a target voltage and back to 0 V. The plate electrode was connected to high potential, while the back electrode was kept on low potential, and the substrate on system ground. The chip is divided into two sensor fields of half the amount of plates each via internal wiring. With this measurement, one single element field of 45 plates was investigated. The C-V-characteristics provide a valuable insight into the static mechanics of the plate. The measurements show the pull-in voltage and the electro-mechanical coupling coefficient. Hysteresis and sticking behavior were observed with a DC sweep from 0 V to a maximal voltage and back to 0 V.

**Figure 8:**
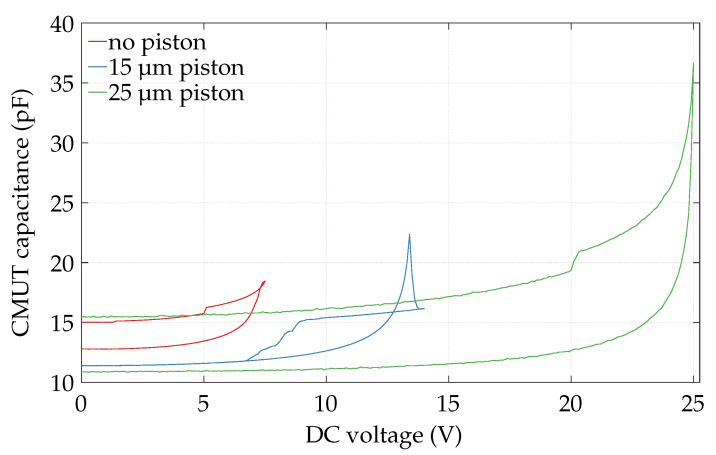
Chip-level measurement of capacitance characteristics in dependence on bias voltage. One element field of 45 plates in parallel is characterized. Measurements show the pull-in voltage and the hysteresis/sticking of the cells of no piston, 15 μm, and 25 μm wide piston.

As depicted in Figure 8, the pull-in voltage rises with the piston diameter. The presented designs have a pull-in of 7.4 V, 13.4 V, and 25.0 V for bare plate, 15 μm piston, and 25 μm piston, respectively. The base capacity at 0 V is 12.79 pF, 11.38 pF, and 10.88 pF, respectively. This is in accordance within −4.2% to +2.7% with the behavior observed and explained in simulations (see Section 3.3). The stiffness of the cell rises with piston diameter, and thus the pull-in voltage. Furthermore, this increase in stiffness lowers the base capacity due to less static displacement from atmospheric pressure. Additionally, hysteresis at decreasing voltage shows sticking of most of the plates after pull-in. The design of a 15 μm piston further reveals no sticking and a peak in capacitance at pull-in, which might relate to anti-sticking bumps arranged on a ring shape on the bottom of the plate. This ring has the same radius as the piston in this case. Those bumps have not been accounted for as no pull-in operation is intended. These phenomena are subject for further investigations.

Additionally, this measurement allows for the calculation of the electro-mechanical coupling coefficient $k^{2}$, according to Fraser et al. based on Berlincourt [30,31,32]. With static and free capacitance extracted from the measurements just below the pull-in voltage, a $k^{2}$ of 0.26, 0.60, and 0.87 is calculated for plate, and smaller and bigger piston, respectively. The piston improves the coupling due to a higher average displacement of the plate, which results in a smaller gap, and hence enhanced electric fields.

### 4.3. Acoustic Characterization

Furthermore, acoustic measurements demonstrate the intended effect of enhanced sound pressure from CMUT piston designs. Here, the CMUT was fixed on a stage facing a hydrophone (2 mm Needle Hydrophone, Precision Acoustics Ltd., Dorchester, UK) in a silicone oil bath. The silicone oil was of relatively low viscosity, with $50\times 10^{-6}\;m^{2}s^{-1}$. The sensors were glued into a premolded cavity package (Infineon PG-DSOF-8-16) with a thin film of epoxy. This provided a sufficiently rigid baffle. The different sensor designs were driven with a 100 ns pulse with an amplitude of 80% of the respective pull-in voltage. The distance between CMUT and hydrophone was 1.17 cm.

**Figure 9:**
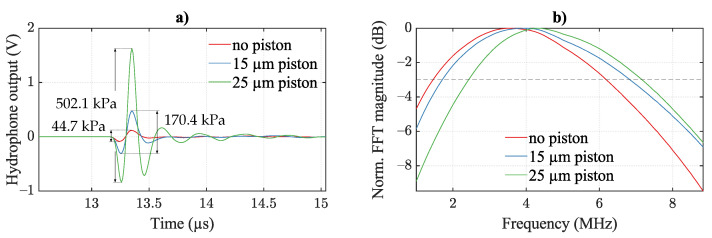
Acoustic measurements of CMUT response to a 100 ns pulse. (**a**) Raw waveform from hydrophone voltage. (**b**) Corresponding Fourier transform of the received waveform including hydrophone calibration. Dotted line indicates −3 dB level for bandwidth evaluation. Surface sound pressure values are noted in (**a**) including hydrophone calibration and diffraction loss. CMUTs are actuated with 90 parallel cells comprising no pistons, and 15 μm and 25 μm wide pistons.

The hydrophone data is depicted in Figure 9a. Comparing bare plate with 15 μm piston, an increase by a factor of 3.8, from 44.7 kPa to 170.4 kPa, is detected in maximum surface sound pressure. Moreover, the improvement from 15 μm piston to 25 μm piston is a factor of 2.9, from 170.4 kPa to 502.1 kPa. Therefore, the maximum demonstrated gain from the addition of a piston is a factor of 11.2, from 44.7 kPa to 502.1 kPa. The maximum surface sound pressure levels are calculated with hydrophone calibration and with accounting for diffraction loss [33,34], neglecting attenuation in the propagation medium. The respective transmit sensitivities are 7.4 kPa/ V, 10.7 kPa/ V, and 24.8 kPa/ V, an enhancement by a factor of 3.35. Furthermore, the Fourier transforms of the waveforms are depicted in Figure 9b with accounting for the hydrophone calibration. These present the frequency characteristics of the sensor designs in silicone oil. Maximum sound pressure is detected at 3.35 MHz, 3.64 MHz, and 4.21 MHz for no piston, 15 μm wide piston, and 25 μm wide piston, respectively. The corresponding ‘-3dB’-bandwidth is 4.5 MHz, 4.93 MHz, and 4.4 MHz. These correspond to a fractional bandwidth of 134.0%, 135.0%, and 106.8%, respectively.

With these measurements, we successfully demonstrate the implementation of the addition of a piston to substantially increase the emitted sound pressure. In immersion, the center frequency rises with the presence/width of a piston. Here, the center frequency depends on displaced mass and the spring constant of the plate. On the one hand, mass rises with the piston due to the piston material mass and the additionally deflected oil (more average displacement). This lowers the center frequency. On the other hand, plate stiffness rises with the piston, increasing the spring constant, and thus increasing center frequency. This dominantly counteracts the mass effect. In immersion, the dominant mass contribution is given by the medium, compared to the vibrometer results in Section 4.1 measured in air (lower mass of medium), where the piston mass has a significant influence on the dynamic mechanics of the transducer.

## 5. Conclusions

With this work, we present a novel design approach for CMUT sensors by implementing an insulator piston on top of a movable plate to introduce piston-like non-flexural displacement for enhanced sound pressure output. Further, a thin plate and small gap are intended to provide low-voltage operation. Thereby, the two major drawbacks of CMUTs, the requirements of high voltages and limited output pressure, are directly addressed. With the help of FEM simulations, a feasible design is determined for the targeted operational regime. Low MHz (<10 MHz) and low-voltage (<25 V) CMUTs without piston and with a small (34% plate coverage) and big piston (57% plate coverage) are fabricated without any additional fabrication steps in the available surface micromachining technology. A proof of concept is given by vibrometer and LCR meter measurements in air, providing mere sensor characteristics. The piston-like movement of the plate is proven. The characterization in silicone oil reveals an improvement in emitted sound pressure by a factor of 11 with the implementation of a piston of 57% plate coverage. This performance increase in operational voltage requirements and emitted sound pressure raises the state of the art and opens new fields of applications for CMUT sensors. Further research will include different cell geometries in terms of plate diameter, plate thickness, and plate coverage by the piston, and will demonstrate applications such as trough-wall fill-level sensing.

## Figures and Tables

**Table 2:** Design parameters of CMUT with piston.

Plate and piston geometry	Circular
Plate thickness *c*	800 nm
Plate diameter *d*	44 μm
Vacuum gap height *h*	120 nm
Vacuum gap insulator height	25 nm
Piston sizes *a*	0, 15, 25 μm
Piston height *b*	2.5 μm
Plate matrix	9 × 10
Active area on chip *x*, *y*	500 × 600 μm^2^ = 0.3 mm^2^
Pitch $p_{x}$; $p_{y}$	61.5μm; 56 μm

**Table 3:** Comparison of resonance frequency and pull-in voltage in simulation and measurement.

	Piston (μm)	Simulation	Measurement	Deviation (%)
Resonance	0	6.52	6.43 ± 0.024	+1.4
frequency (MHz)	15	5.22	5.11 ± 0.012	+2.1
	25	6.32	6.34 ± 0.004	−0.3
	0	7.60	7.4 ± 0.1	+2.7
Pull-in voltage (V)	15	12.85	13.4 ± 0.1	−4.2
	25	24.63	25.0 ± 0.1	−1.5

## Data Availability

Data available on request due to restrictions e.g., privacy or ethical. The data presented in this study are available on request from the corresponding author. The data are not publicly available due to eventual future commercial interests.
